# Relation of corona-specific health literacy to use of and trust in information sources during the COVID-19 pandemic

**DOI:** 10.1186/s12889-021-12271-w

**Published:** 2022-01-06

**Authors:** Saskia Maria De Gani, Fabian Marc Pascal Berger, Elena Guggiari, Rebecca Jaks

**Affiliations:** 1 Careum Foundation, Health Literacy Department, Pestalozzistrasse 3, 8032 Zurich, CH Switzerland; 2 Careum School of Health, Research Departmen, Gloriastrasse 18a, 8006 Zurich, CH Switzerland

**Keywords:** Health literacy, Coronavirus, COVID-19, Infodemic, Information sources, Trust, Use of media, Information seeking behavior, Health information

## Abstract

**Background:**

COVID-19 has developed into a worldwide pandemic which was accompanied by an «infodemic» consisting of much false and misleading information. To cope with these new challenges, health literacy plays an essential role. The aim of this paper is to present the findings of a trend study in Switzerland on corona-specific health literacy, the use of and trust in information sources during the COVID-19 pandemic, and their relationships.

**Methods:**

Three online surveys each with approximately 1′020 individuals living in the German-speaking part of Switzerland (age ≥ 18 years) were conducted at different timepoints during the COVID-19 pandemic, namely spring, fall and winter 2020. For the assessment of corona-specific health literacy, a specifically developed instrument (HLS-COVID-Q22) was used. Descriptive, bivariate, and multivariate data analyses have been conducted.

**Results:**

In general, a majority of the Swiss-German population reported sufficient corona-specific health literacy levels which increased during the pandemic: 54.6% participants in spring, 62.4% in fall and 63.3% in winter 2020 had sufficient corona-specific health literacy. Greatest difficulties concerned the appraisal of health information on the coronavirus. The most used information sources were television (used by 73.3% in spring, 70% in fall and 72.3% in winter) and the internet (used by 64.1, 64.8 and 66.5%). Although health professionals, health authorities and the info-hotline were rarely mentioned as sources for information on the coronavirus, respondents had greatest trust in them. On the other hand, social media were considered as the least trustworthy information sources. Respondents generally reporting more trust in the various information sources, tended to have higher corona-specific health literacy levels.

**Conclusions:**

Sufficient health literacy is an essential prerequisite for finding, understanding, appraising, and applying health recommendations, particularly in a situation where there is a rapid spread of a huge amount of information. The population should be supported in their capability in appraising the received information and in assessing the trustworthiness of different information sources.

## Background

Since the 1960s, different coronaviruses have been discovered, including those causing severe acute respiratory syndrome (SARS) [1, 2]. The new «SARS-CoV-2» (Severe Acute Respiratory Syndrome Coronavirus 2) also belongs to this virus family and was first discovered in Wuhan, China in December 2019. The thereby caused disease was officially named «COVID-19» by the World Health Organization (WHO) on February 11, 2020 [3] and has rapidly developed into a worldwide pandemic. To contain the pandemic, drastic protective measures have been taken by the worldwide affected countries and preventive behavior has been demanded from the population. During the first and second waves of the COVID-19 pandemic, Switzerland was among the countries with the highest numbers of COVID-19 cases per capita in the world [4, 5].

An important step in containing the spread of the virus and reducing the burden on the health system whilst at the same time protecting vulnerable people, is regular and comprehensive communication of information to the population [6]. Since the outbreak of the pandemic, a large amount of information on the virus, the disease, the risks of infection as well as on preventive measures has been produced and disseminated. Not only the need for information has been elevated, but also the supply of information itself. The lack of knowledge about this new virus as well as the threat to health and other areas of society (e.g., economy) brought a flood of constantly changing, sometimes contradictory information. In other words, the coronavirus pandemic was accompanied by an «infodemic» which emerges by a rapid spread of a big amount of valid and invalid information through different communication technologies [7, 8, 6]. As a consequence, the particular challenge to distinguish trustworthy health information from false and misinformation, which can be defined as “fake, unreliable, or not scientifically validated written material regardless of intentional authorship” [9], has strongly increased [10]. Misinformation plays an important role in public health and has created uncertainty also before the COVID-19 pandemic (e.g. during the first stages of the HIV epidemic or during the avian influenza H5N1 outbreak in 2004) [9]. Furthermore, false and misleading information can spread rapidly on social media, in political rhetoric, in general references or at the dinner table which makes the assessment of the reliability of the information even more difficult. To be able to cope with the challenges posed by the «infodemic» as well as the pandemic itself, health literacy plays an essential role. Health literacy (HL) in general encompasses people’s knowledge, motivation and competences to find, understand, appraise and apply health information, make informed decisions for their health and act accordingly [8]. Hence, HL can help the population to access, navigate and understand information on COVID-19, distinguish between reliable and mis-information, and empowers people to make informed health decisions based thereon [6].

In the context of the COVID-19 pandemic, also health information-seeking behavior plays a critical role. Generally, it describes an active and purposeful behavior undertaken by an individual with the objective of finding information on health issues [11]. To date, few studies have analyzed the use of different media during the COVID-19 pandemic. In Greece for example, people searched for information on the COVID-19 pandemic mainly in television, electronic press and news websites, and made only limited use of social media [12]. The authors supposed that this could be due to the raised awareness of the spread of fake news via social media. In the US, the most used information sources during the COVID-19 pandemic were mass media sources such as television, radio, podcasts, or newspapers, whilst the largest online information source were government websites [13]. Regarding the impact on risk perception of the different information sources used by the US population, it was reported that those with a heavy reliance on television and internet as information sources indicated higher levels of general risk perception and protective behavior [14]. Moreover, in order to make crisis and risk communication more effective, it is important to understand where people look for information and how this information shapes their perceptions and actions [14]. Along with the information sources used, the trust in these different sources and the consequences thereof should also be considered: When information is inconsistent, trust tends to decrease [15, 16]. Thus, especially in public health emergencies like the COVID-19 pandemic, it is crucial to provide clear and consistent information [15] to build trust and promote adapted behavior.

Relating to the use of and trust in health information sources and its consequences, the question that arises is how they are associated with HL. Understanding which health information sources are used and trusted by people with limited HL levels could help to identify support strategies specifically for these people that often report higher health risks as well. A previous study found that people with lower HL are more likely to mistrust information from specialist doctors and dentists and report more negative perceptions regarding their health care experience. They are also more likely to prefer health information from social media, blogs or celebrity webpages [17].. Additionally, a qualitative study on the relationship between patients and health care providers found that trust seems to be play an important role in the treatment of HIV. Therefore, a trustworthy relationship between patients and health care providers seems to be an additional and important aspect regarding HL [18]. In addition, the COVID-19 pandemic and the related «infodemic» [7] have made HL a highly relevant issue in the context of infectious diseases and highlight again that HL as a relational construct depends not only on the individual resources but also on the demands of social systems [19, 20]. However, so far only few studies have assessed HL in the context of infectious diseases [21], and to our knowledge only few countries have measured corona-specific HL, as for example Germany [6, 22]. Therefore, the aim of this study was (1) to assess corona-specific HL of the German-speaking population of Switzerland, (2) to examine their health information-seeking behavior and trust in information sources, (3) to explore differences in their use of and trust in information sources in relation to corona-specific HL during the COVID-19 pandemic, and (4) to investigate how trust in different information sources affects corona-specific HL.

## Methods

A total of three online surveys was conducted among the German-speaking population of Switzerland to investigate their corona-specific HL levels at different timepoints of the COVID-19 pandemic and when different preventative measures were put in place. These three timepoints were in May (spring), September/October (fall) and November/December (winter) 2020. The three samples included 1′020 persons (age ≥ 18 years) in each case and were all representative for the population of German-speaking Switzerland. Data were collected by means of computer-assisted web interviews (CAWI).

The first survey on corona-specific HL in Switzerland was conducted by the Careum Foundation (Switzerland) on behalf of the Swiss Federal Office of Public Health (FOPH) as supplementary mandate to the « Health Literacy Survey Switzerland 2019-2021» (not yet published). Gallup AG Switzerland was commissioned to collect the data of the first survey. The two further surveys were conducted in cooperation with the University of Bielefeld (Germany) and Gesundheit Österreich GmbH (Austria) as well as the Health Department of the Canton of Zurich (Switzerland) and on behalf of the Federal Ministry of Health Germany. Data collection of these two surveys were carried out by the Institut für Demoskopie Allensbach (Germany). Data analyses for all of the three surveys was carried out by gfs.bern AG (Switzerland) and Careum Foundation (Switzerland).

**Fig. 1:**
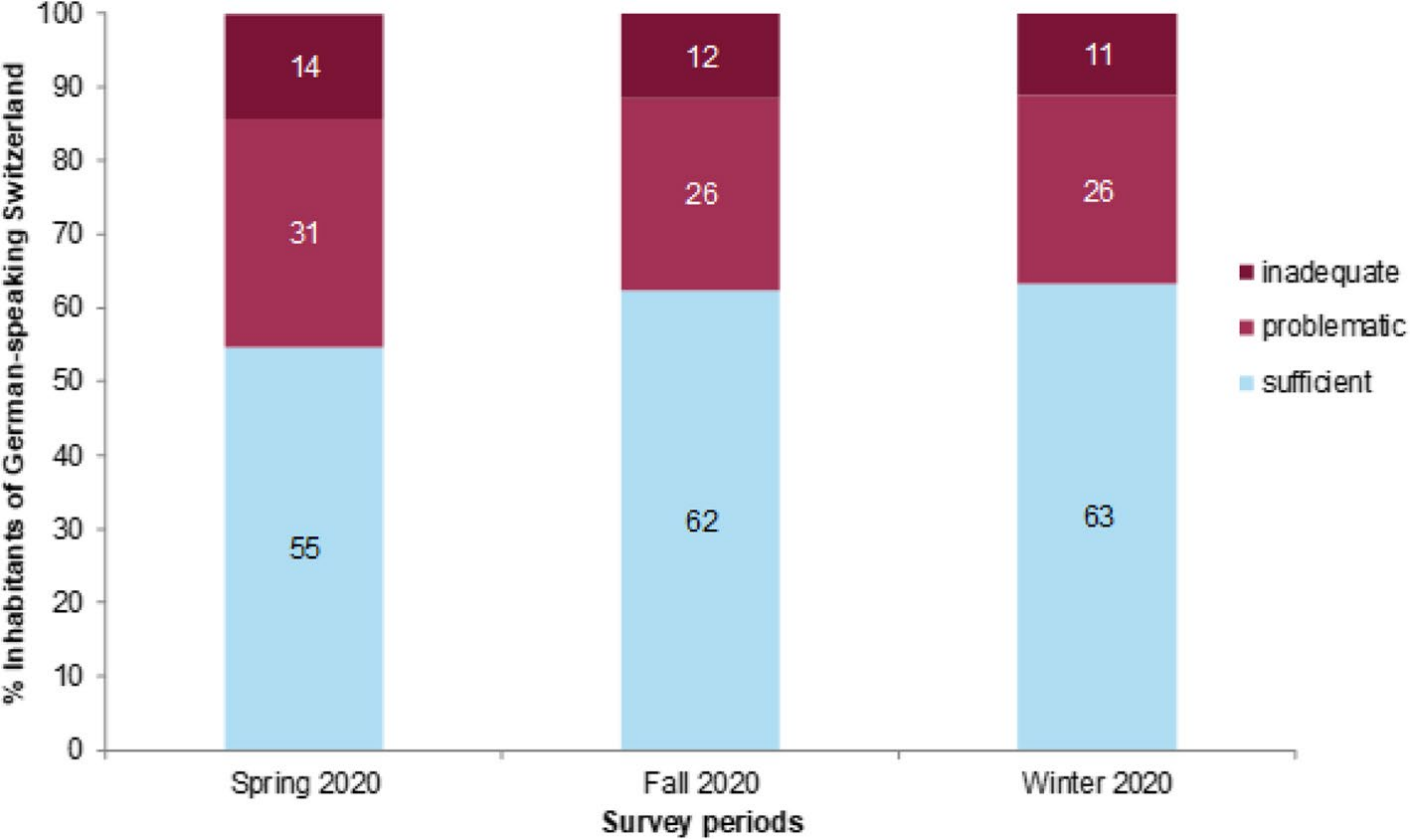
Index of corona-specific health literacy. Legend: Fig. 1 describes the amount of inadequate, problematic or sufficient corona-specific health literacy among the German-speaking population in Switzerland over all three survey periods

Partial results of the study were published in German as a final report, focusing on sample description (Table 3), corona-specific health literacy (Fig. 1, Table 4, 5, 6 and 7) and the use of information sources (Table 8) [23]. To deepen our understanding of the relationship between corona-specific health literacy, use of information source and trust in information source, we conducted further statistical analysis using bivariate and multivariate methods like correlation coefficient spearman rho, principal component analysis and multiple regression analysis, which are only included in the present manuscript.

### Questionnaire

In order to assess corona-specific HL of the Swiss-German population, a specifically developed instrument with 22 items (HLS-COVID-Q22) was used. It is based on the existing and widely used survey instruments to assess general health literacy, the HLS-EU-Q16 and HLS-EU-Q47 [22]. The HLS-COVID-Q22 has proven to be a reliable instrument for measuring corona-specific HL and has a high internal consistency (α = 0.940; *p* = 0.891) [22]. The HLS-COVID-Q22 includes questions on (1) feeling informed, (2) feeling confused, (3) corona-specific HL, (4) information sources and (5) their trustworthiness, (6) information behavior, (7) preventive behavior and (8) socio-demographic aspects of survey participants. In the second (September/October 2020) and third (November/December 2020) survey waves, additional items were collected, such as subjective assessment of health and quality of life, prospects, worries and fears, knowledge on the coronavirus and knowledge and attitudes on vaccination, as well as on the propensity to vaccinate when a COVID-19 vaccine would be available. These questionnaires were developed in cooperation with the above-mentioned colleagues from Germany and Austria and thereupon specifically adapted to the Swiss setting.

Corona-specific HL items could be answered with «very difficult» (= 1), «difficult» (= 2), «easy» (= 3) or «very easy» (= 4). To assess corona-specific HL, a mean value was calculated from the values of the 22 questions. According to Okan et al. [6, 22], the following cut-off values and levels were applied:

- mean value ≤2.5: «insufficient corona-specific health literacy»,
- mean value > 2.5 - ≤ 3.0: «problematic corona-specific health literacy»,
- mean value ≥3.0: « sufficient corona-specific health literacy».

Only cases that answered at least 80% of the items were considered in the analyses.

#### Statistical analyses

Descriptive, bivariate, and multivariate data analyses were conducted using IBM SPSS Statistics v.26 software (IBM Corp. Armonk, NY, 182 USA). Samples were combined across all three measurement points and analyzed as one large data set. The advantage of this approach is that more reliable statements can be made on population-specific differences. For the bivariate analysis, Pearson’s chi-square, Spearman’s rank correlation coefficient, and regression analysis were used. The results were considered statistically significant based on the threshold value *p ≤* 0.05.

To reduce the dimensionality and increase the interpretability of the data regarding trust in the information sources, a principal component analysis (PCA) was carried out (Table 1). To determine the number of component loadings, eigenvalue greater than 1 were used.

**Table 1:** PCA – overall trust in information sources: total variance explained

	Initial Eigenvalues			Extraction Sums of Squared Loadings			Rotation Sums of Squared Loadings		
Component	Total	% of Variance	Cumulative %	Total	% of Variance	Cumulative %	Total	% of Variance	Cumulative %
1		43.813	43.813	5.696	43.813	43.813	5.161	39.698	39.698
2	1.862	14.326	58.139	1.862	14.326	58.139	2.397	18.441	58.139
3	0.960	7.386	65.525						
4	0.761	5.855	71.379						
5	0.696	5.352	76.731						
6	0.599	4.606	81.337						
7	0.510	3.924	85.261						
8	0.437	3.358	88.619						
9	0.368	2.827	91.446						
10	0.350	2.696	94.142						
11	0.315	2.421	96.562						
12	0.241	1.853	98.415						
13	0.206	1.585	100.000						

*Note: Extraction method: principal component analysis*

Based on the item loading on a component, each component was interpreted and item loadings of 0.40 or higher were retained [24] (Table 2). The PCA led to two new variables: general sources (factor 1) and social sources (factor 2) which cumulatively explain 58% of the variance.

**Table 2:** PCA – overall trust in information sources: rotated component matrix

	Component^a^	
	Factor 1: general sources	Factor 2: social sources
Television	0.750	
Teletext	0.665	
Radio	0.774	
Internet	0.556	
Social Media		0.833
Messenger		0.810
Newspaper - Offline	0.720	
Newspaper - Online	0.709	
News App	0.603	0.410
Info-Hotline	0.793	
Health Professionals	0.749	
Health Authorities	0.803	
Family, Friends, Colleagues, Acquaintances		0.564

*Note: Extraction method: principal component analysis*

*Rotation method: Varimax with Kaiser Normalization*

*a. Rotation converged in 3 iterations*

Based on the PCA, four indices were created to distinguish between *overall trust* and *actual trust* in information sources. *Overall trust* corresponds to trust in information and media sources irrespective of their use, *actual trust* describes trust in information sources and media which were effectively used (filtered by a filter variable). Index 1 (*overall trust*) and index 3 (*actual trust*) include the variables from factor 1 «general sources», whereas index 2 (*overall trust*) and 4 (*actual trust*) include the variables form factor 2 «social sources», except for the variable «News App» which was assigned to the «general sources» because of the higher loading to factor 1 and the fit of content. The index values result from the mean value of the associated items ranging from «very trustworthy» (= 3) to «not trustworthy at all» (= 0). The index values were normalized to 100. To correlate the indices concerning trust in information sources to corona-specific HL, Spearman’s rho was considered. To further evaluate how trust in information sources affects corona-specific HL, a multiple regression analysis was conducted. Effect sizes (sr) were evaluated according to Cohen [25]:

- sr = 0.10 corresponds to a weak effect,
- sr = 0.30 corresponds to a medium effect,
- sr = 0.50 corresponds to a strong effect.

## Results

### Sample characteristics

Slightly more women (50.4%) responded to the questionnaire. Mean age of all respondents was 46.2 (± 16.9) years, whereby 85.2% of the respondents were born in Switzerland (Table 3). The majority (88.8%) reported an upper secondary level education or higher, and one fifth (20.5%) an education background in the health sector.

**Table 3:** Characteristics of study participants

	Spring 2020	Fall 2020	Winter 2020
	n (%)	n (%)	n (%)
**Total, N (%)**	1012 *(100.0)*	1026 *(100.0)*	1018 *(100.0)*
**Gender**			
Male	498 *(49.2)*	509 *(49.6)*	510 *(50.1)*
Female	514 *(50.8)*	517 *(50.4)*	508 *(49.9)*
**Age**			
18-29 years old	185 *(18.3)*	197 *(19.2)*	220 *(21.6)*
30-39 years old	164 *(16.2)*	181 *(17.6)*	224 *(22.0)*
40-49 years old	186 *(18.4)*	193 *(18.8)*	154 *(15.1)*
50-59 years old	179 *(17.7)*	196 *(19.1)*	190 *(18.7)*
60-69 years old	175 *(17.3)*	167 *(16.3)*	157 *(15.4)*
70 years old or older	108 *(10.7)*	92 *(9.0)*	73 *(7.2)*
**Born in Switzerland**^a^			
Yes	868 *(85.8)*	885 *(86.3)*	851 *(83.6)*
No	135 *(13.3)*	141 *(13.7)*	167 *(16.4)*
**Education**			
Compulsory school	100 *(9.9)*	125 *(12.2)*	118 *(11.6)*
Upper secondary level II	491 *(48.5)*	401 *(39.1)*	410 *(40.3)*
Tertiary level	421 *(41.6)*	500 *(48.7)*	490 *(48.1)*
**Education in the health sector**^a^			
Yes	202 *(20.0)*	195 *(19.0)*	228 *(22.4)*
No	796 *(78.7)*	831 *(81.0)*	790 *(77.6)*
**Employment status**			
Employed	522 *(51.6)*	549 *(53.5)*	567 *(55.7)*
Self-employed	94 *(9.3)*	83 *(8.1)*	96 *(9.4)*
Unemployed	52 *(5.1)*	51 *(5.0)*	49 *(4.8)*
Retired	191 *(18.9)*	187 *(18.2)*	156 *(15.3)*
Student	55 *(5.4)*	69 *(6.7)*	61 *(6.0)*
Other	98 *(9.7)*	87 *(8.5)*	89 *(8.7)*
**Net household income**^a^			
Less than CHF 4′000	243 *(24.0)*	308 *(30.0)*	279 *(27.4)*
CHF 4′000 - CHF 6′000	214 *(21.1)*	245 *(23.9)*	247 *(24.3)*
CHF 6′001 - CHF 8′000	159 *(15.7)*	200 *(19.5)*	204 *(20.0)*
CHF 8′001 - CHF 10′000	85 *(8.4)*	124 *(12.1)*	138 *(13.6)*
CHF 10′001 - CHF 12′000	60 *(5.9)*	69 *(6.7)*	73 *(7.2)*
More than CHF 12′000	48 *(4.7)*	80 *(7.8)*	77 *(7.6)*
**Chronic illnesses**^a^			
Yes, several	231 *(22.8)*	145 *(14.1)*	128 *(12.6)*
Yes, one	116 *(11.5)*	340 *(33.1)*	305 *(30.0)*
No	626 *(61.9)*	541 *(52.7)*	585 *(57.5)*

*a. Due to missing answers, the sum may be less than 100%*

*Corona-specific health literacy.*

More than half of the German-speaking population of Switzerland reported sufficient corona-specific HL levels. The levels increased from spring to fall and then stagnated until winter (Fig. 1). Over all three timepoints, at least around one third of the respondents reported inadequate or problematic corona-specific HL. The differences between time periods were statistically relevant (*p* ≤ 0.05).

Women tended to have slightly higher corona-specific HL levels than men (62.9% vs. 57.6%). In addition, for men, the proportion with inadequate corona-specific HL (14.2%) was slightly higher than for women (10.4%).

The German-speaking population reported little difficulties in *finding information* on the coronavirus and COVID-19 (Table 4). For all three timepoints, it seemed easy or very easy for the majority (84, 85, 84%) to find information on the coronavirus or on preventive behavior on the internet, in newspapers, magazines or on TV. More difficulties were reported relating to finding information on potential infection, where to get professional help and how vulnerable they were in case of an infection. However, as the pandemic progressed, respondents reported less difficulties in dealing with these issues.

**Table 4:** Finding information on the coronavirus and COVID-19

	*% Inhabitants of German-speaking Switzerland*				
	Very easy	Easy	Difficult	Very difficult	*p-value*
**To find information on the internet**					*0.539*
Spring 2020	49%	42%	7%	2%	
Fall 2020	51%	42%	6%	1%	
Winter 2020	51%	41%	6%	2%	
**To find information on the internet about behaviors that can prevent the infection**					*0.197*
Spring 2020	44%	47%	8%	1%	
Fall 2020	49%	43%	7%	1%	
Winter 2020	50%	42%	7%	1%	
**To find out where to get professional help in case of an infection with the coronavirus**					*< 0.001*
Spring 2020	28%	51%	17%	4%	
Fall 2020	36%	48%	14%	2%	
Winter 2020	40%	45%	13%	2%	
**To find information in newspapers, magazines, or TV on behaviors to avoid infection**					*0.080*
Spring 2020	31%	53%	13%	4%	
Fall 2020	34%	51%	13%	2%	
Winter 2020	37%	47%	14%	2%	
**To find information on a potential infection**					*< 0.001*
Spring 2020	21%	50%	21%	8%	
Fall 2020	31%	47%	19%	3%	
Winter 2020	36%	45%	16%	3%	
**To find information on personal vulnerability**					*0.013*
Spring 2020	25%	48%	22%	5%	
Fall 2020	29%	45%	22%	4%	
Winter 2020	32%	46%	18%	4%	

For a large proportion of the respondents, the various information on the coronavirus or COVID-19 was easy or very easy to *understand* (Table 5). In spring 2020, 90% of the respondents found it (very) easy to understand hygiene instructions from the FOPH. This result hardly changed as the pandemic progressed. Also stable remained the difficulties concerning the understanding of instructions from specialists on protective measures, of advices on protective measures from family members and friends and information from the media on protection against an infection. More difficulties were reported in understanding of information from the internet, newspapers, and TV on the risks of the coronavirus, although for a large proportion of respondents this was still considered (very) easy (78% vs. 82% vs. 81%).

**Table 5:** Understanding the information on the coronavirus and COVID-19

	*% Inhabitants of German-speaking Switzerland*				
	Very easy	Easy	Difficult	Very difficult	*p-value*
**Understand hygiene instructions from the Federal Office of Public Health (FOPH)**					*0.077*
Spring 2020	47%	43%	8%	2%	
Fall 2020	49%	40%	8%	3%	
Winter 2020	48%	39%	9%	4%	
**Understand instructions from specialists on protective measures**					*< 0.001*
Spring 2020	32%	55%	11%	2%	
Fall 2020	40%	48%	10%	3%	
Winter 2020	42%	45%	10%	4%	
**Understand advice on protective measures from family members and friends**					*< 0.001*
Spring 2020	31%	54%	12%	3%	
Fall 2020	39%	48%	11%	2%	
Winter 2020	39%	47%	12%	2%	
**Understand information from the media on protection against an infection**					*0.038*
Spring 2020	31%	54%	12%	3%	
Fall 2020	36%	50%	11%	3%	
Winter 2020	38%	47%	11%	4%	
**Understand information from the internet on the risks of the coronavirus**					*0.002*
Spring 2020	25%	55%	17%	3%	
Fall 2020	32%	51%	15%	2%	
Winter 2020	33%	48%	16%	3%	
**Understand information in newspapers and on TV on the risks of the coronavirus**					*0.001*
Spring 2020	23%	55%	19%	3%	
Fall 2020	28%	54%	16%	2%	
Winter 2020	31%	50%	16%	3%	

Compared to finding and understanding information on the coronavirus and COVID-19, *appraising* this information seemed more difficult (Table 6). For one fifth of the respondents, it was difficult to assess which protective measures and behavioral instructions should be taken in order to protect themselves from an infection. However, this became easier as the pandemic progressed (20% vs. 14%). Similar findings were found in assessing personal risks. Difficulties in assessing a possible infection also decreased in fall and winter 2020 compared to the beginning of the pandemic, when almost half of the respondents reported difficulties. In spring 2020, only 45.7% of respondents found it (very) easy to assess the trustworthiness of information from the media. This seemed to become easier as the pandemic progressed. Nevertheless, almost half of the respondents (46%) still had troubles with it in winter 2020.

**Table 6:** Appraising the information on the coronavirus and COVID-19

	*% Inhabitants of German-speaking Switzerland*				
	Very easy	Easy	Difficult	Very difficult	*p-value*
**To assess which protective measures to take against the coronavirus**					*< 0.001*
Spring 2020	30%	50%	16%	4%	
Fall 2020	41%	42%	14%	3%	
Winter 2020	40%	46%	12%	2%	
**To assess which behaviors are linked to an increased risk of infection**					*< 0.001*
Spring 2020	24%	52%	19%	5%	
Fall 2020	32%	47%	18%	3%	
Winter 2020	33%	43%	20%	4%	
**To assess personal risk**					*0.001*
Spring 2020	24%	47%	25%	4%	
Fall 2020	30%	41%	25%	4%	
Winter 2020	32%	44%	20%	4%	
**To assess whether being infected with the coronavirus**					*< 0.001*
Spring 2020	16%	38%	33%	13%	
Fall 2020	24%	37%	33%	6%	
Winter 2020	26%	40%	29%	5%	
**To assess the trustworthiness of information from the media**					*< 0.001*
Spring 2020	13%	32%	36%	19%	
Fall 2020	19%	34%	34%	13%	
Winter 2020	19%	35%	30%	16%	

*The application of coronavirus-related information* on one’s own life was also reported to be more difficult compared to finding and understanding such information (Table 7). Still, for a large proportion of respondents in spring 2020, it was (very) easy to behave in a way that does not infect others (84%), to follow the instructions from professionals to protect themselves against an infection (86%) and to apply the information received by professionals to decide how to deal with an infection (79%). Applying such information became slightly easier as the pandemic progressed. It was somewhat more difficult for the respondents to decide based on information from the media on how to protect themselves from an infection or how to deal with an infection.

**Table 7:** Applying information on the coronavirus and COVID-19

	*% Inhabitants of German-speaking Switzerland*				
	Very easy	Easy	Difficult	Very difficult	*p-value*
**To behave in a way that does not infect others**					*0.002*
Spring 2020	36%	48%	14%	2%	
Fall 2020	42%	46%	10%	2%	
Winter 2020	44%	43%	10%	3%	
**To follow the instructions from professionals to protect themselves against an infection**					*0.001*
Spring 2020	32%	54%	12%	2%	
Fall 2020	41%	48%	9%	2%	
Winter 2020	40%	47%	10%	3%	
**To apply the information received by professionals to decide how to deal with an infection**					*< 0.001*
Spring 2020	25%	54%	19%	2%	
Fall 2020	32%	52%	13%	3%	
Winter 2020	36%	48%	13%	3%	
**To decide based on information from the media, how to protect themselves from an infection**					*< 0.001*
Spring 2020	25%	57%	14%	4%	
Fall 2020	32%	48%	16%	4%	
Winter 2020	34%	49%	12%	5%	
**To decide based on information from the media, how to deal with an infection**					*< 0.001*
Spring 2020	18%	49%	27%	6%	
Fall 2020	27%	45%	23%	5%	
Winter 2020	27%	44%	23%	6%	

### Use of and trust in information sources

During the COVID-19 pandemic, the most used media was – for all three timepoints – the television, followed by the Internet. All other information sources were used by less than half of the respondents (Table 8). The proportion of individuals using a certain information source remained more or less stable during the pandemic.

**Table 8:** Proportion of individuals using a certain information source

*Information sources*^*a*^	*% Inhabitants of German-speaking Switzerland*			*p-value*
	Spring 2020	Fall 2020	Winter 2020	
Television	73%	70%	72%	*0.237*
Internet	64%	65%	67%	*0.494*
Health authorities	42%	44%	41%	*0.465*
Radio	41%	36%	29%	*0.066*
Newspaper - Offline	35%	35%	33%	*0.746*
Family, friends, acquaintances	34%	34%	34%	*< 0.001*
News App	27%	27%	25%	*0.462*
Health professionals	22%	26%	29%	*0.001*
Social media	26%	24%	27%	*0.177*
Info-Hotline	3%	6%	5%	*0.034*

*a. Not all sources of information asked are reported in the table*

Although health professionals, health authorities and the info-hotline have been rarely mentioned as sources of information to find information about the coronavirus, respondents reported to have greatest trust in these sources (Table 12, see Appendix). The television was considered trustworthy by most of the respondents, similarly to the internet. Social media was considered as the least trustworthy source of information: less than one fourth of the respondents reported to trust this kind of information source.

### Use of information sources and trust in it by corona-specific health literacy level

Respondents with a sufficient level of corona-specific HL tended to use more often the TV and the internet, as well as health professionals and health authorities (e.g., FOPH and Cantonal Health Departments) as information sources than people with problematic or inadequate corona-specific HL (Table 9). Social media is used in an equal amount by all of the respondents, independent of the corona-specific HL level.

**Table 9:** Proportions of participants using different information sources by corona-specific HL

	*% Inhabitants of German-speaking Switzerland*			
*Information sources*^*a*^	corona-specific HL			*p-value*
	inadequate	problematic	sufficient	
Television	63%	73%	74%	*< 0.001*
Internet	58%	66%	67%	*0.004*
Radio	35%	40%	40%	*0.178*
Health authorities	33%	40%	46%	*< 0.001*
Family, friends, acquaintances	32%	34%	35%	*0.504*
Newspaper - Offline	31%	34%	36%	*0.112*
Social media	26%	25%	26%	*0.712*
News App	23%	24%	28%	*0.022*
Health professionals	20%	24%	28%	*0.004*
Info-Hotline	4%	5%	5%	*0.521*

*a. Not all sources of information asked are reported in the table*

Furthermore, respondents who trusted a specific information source more often, tended to have higher corona-specific HL (Table 10). This is true for all the information sources: People with a sufficient corona-specific HL stated significantly more often that health professionals and health authorities were very trustworthy sources of information, compared to those with inadequate or problematic corona-specific HL.

**Table 10:** Proportions of participants trusting the different information sources by corona-specific HL

	*% Inhabitants of German-speaking Switzerland*						
*Information sources*^*a*^	corona-specific HL						*p-value*
	inadequate		problematic		sufficient		
	++	+	++	+	++	+	
Health professionals	19%	54%	33%	55%	50%	42%	*< 0.001*
Health authorities	19%	42%	33%	49%	53%	36%	*< 0.001*
Info-Hotline	14%	48%	31%	54%	46%	41%	*< 0.001*
Television	5%	44%	9%	62%	20%	60%	*< 0.001*
Internet	5%	43%	9%	61%	22%	57%	*< 0.001*
Family, friends, acquaintances	5%	35%	5%	46%	12%	49%	*< 0.001*
Radio	4%	42%	8%	64%	20%	60%	*< 0.001*
Newspaper - Offline	4%	34%	4%	57%	14%	58%	*< 0.001*
News App	2%	26%	4%	43%	10%	52%	*< 0.001*
Social media	2%	12%	3%	12%	5%	20%	*< 0.001*

*a. Not all sources of information asked are reported in the table*

*++ = very trustworthy; + = rather trustworthy*

### Correlation

Based on the findings of the PCA and the derived indices, respondents reported most trust in *general sources* which they had actually used (actual trust: M = 66.55, SD = 20.11). The trust-level of *general sources* of information, regardless of their use was lower (overall trust: M = 61.83, SD = 18.39). *Social sources* were also more trusted when they were actually used (actual trust: M = 49.70, SD = 25.38 vs. overall trust: M = 39.29, SD = 20.68).

Results of the Spearman’s correlation show that respondents with higher corona-specific HL had higher trust in *general sources*, independent of their use, and reported higher trust in *social sources* they had actually used compared to the overall trust in *social sources* (Table 11). However, the effects found regarding trust in *social sources* (index 2 and 4) only show weak effects (sr = 0.165 vs. sr = 0.235).

**Table 11:** Indices of general/social sources and corona-specific HL

			Index 1: overall trust in «general sources»	Index 2: overall trust in «social sources»	Index 3: actual trust in «general sources»	Index 4: actual trust in «social sources»	Corona-specific HL
Spearman’s Rho	Index 1: overall trust in «general sources»	Correlation coeff.	1	0.281^*^	0.758^*^	0.272^*^	0.383^*^
		N	3131	3131	3078	1307	3077
	Index 2: overall trust in «social sources»	Correlation coeff.	0.281^*^	1	0.193^*^	0.662^*^	0.165^*^
		N	3131	3131	3078	1307	3077
	Index 3: actual trust in «general sources»	Correlation coeff.	0.758^*^	0.193^*^	1	0.316^*^	0.336^*^
		N	3078	3078	3078	1265	3032
	Index 4: actual trust in «social sources»	Correlation coeff.	0.272^*^	0.662^*^	0.316^*^	1	0.235^*^
		N	1307	1307	1265	1307	1296
	Corona-specific HL	Correlation coeff.	0.383^*^	0.165^*^	0.336^*^	0.235^*^	1
		N	3077	3077	3032	1296	3077

* The correlation is significant at the 0.01 level (two-sided)

The regression analysis (ANOVA), which examined the relationship between the dependent variable «corona-specific HL» and the four indices, indicated significant differences in corona-specific HL between respondents trusting *general sources* and *social sources*. This is true for overall trust (*F* (2, 3013) = 245.417, *p* <  0.001) as well for actual trust (*F* (2, 1213) = 72.688, *p* <  0.001).

Based on these calculations the following regression equations result (*p* > 0.001):

1. The regression equation for overall trust is:
  $$corona- specific\ HL=2.418+0.010\ \left( general\ sources\right)+0.002\ \left( social\ sources\right)$$
2. The regression equation for actual trust is:
  $$corona- specific\ HL=2.556+0.006\ \left( general\ sources\right)+0.003\ \left( social\ sources\right)$$

The t-test for all regression coefficients and constants report a significance level of *p* <  0.001.

The results of both regressions suggest that a higher level of trust in *general sources* and *social sources* results in a higher level of corona-specific HL. However, regarding overall trust, its effect regarding *general sources* is five time higher than its effect regarding *social sources*. When considering actual trust, the effect is weaker but still noteworthy. 14% (R^2^ = 0.140) of the dispersion in the overall trust model and 10.6% (R^2^ = 0.106) percent in the actual trust model of health literacy is explained by the two independent variables. In addition, both regression models suggest a medium effect between overall trust [ *f*^2^ = 0.16 ] and a weak effect between actual trust [ *f*^2^ = 0.12 ] in information source and corona-specific HL [25].

## Discussion

This study presents the first results on corona-specific HL among the German-speaking population in Switzerland, using the adapted HLS-EU-Q22 to assess corona-specific HL. The results show that the German-speaking population in Switzerland in general reports sufficient corona-specific HL levels. Their ability in finding, understanding, appraising, and applying corona-specific health information even increased during the pandemic: 54.6% participants in spring 2020, 62.4% in fall and 63.3% in winter 2020 reported sufficient corona-specific HL. Most difficulties concerned the appraisal and application of health information on the coronavirus on daily life and special health-related tasks. In addition, the corona-specific HL seems to be slightly higher than the general HL of the Swiss population. A study conducted in 2015 showed that 46% of the respondents had a sufficient general HL [26]. Similar results were reported in Germany, where the corona-specific HL was also higher than the general HL [6, 22]. One explanation for this difference between corona-specific and general HL as well as the increase of corona-specific HL during the pandemic might be the radical change in the everyday life of the population brought by the pandemic, where constant and detailed information on the latest developments have been provided by the health authorities and media. They provided a wide range of corona-specific information for the population and communicated the preventive measures that needed to be implemented to protect from the coronavirus. Furthermore, the information provided was very specific to this health issue. Moreover, the pandemic seemed to have changed the information behavior of the population: During the entire course of the pandemic, the population sought information on health topics more frequently than before the pandemic [23, 22]. Nevertheless, a significant proportion of the German-speaking population still had an inadequate or problematic corona-specific HL, and thus often difficulties with handling corona-related health issues. Regarding the containment of the current pandemic and the application of the needed preventive measures by the population, it is of great importance to strengthen their corona-specific and general HL.

In this regard and in terms of the increasing importance of online health information, it has to be kept in mind that accessing valid and trustworthy health information on the internet represents a big challenge [27] especially for people with low HL [14, 28, 29]. Online health information differs considerably in reliability and incorrect or misleading information are quite common [30, 27]. Hence, the population is constantly exposed to a huge amount of information with poor quality which holds also true for the corona pandemic. People facing problems with accessing and assessing the quality of online information are more prone to trust false information. Since the internet is a widely used source for the access of health information, it is important that the population and especially those with low corona-specific and general HL are adequately supported to distinguish between correct and incorrect information. Especially during a public health crisis like the corona pandemic, it is important that the population has the knowledge and competencies to handle health information appropriately and to understand which information is reliable to being able to follow and apply the officially promoted preventive public health recommendations.

The present findings show that during the COVID-19 pandemic not only the internet but by far the most used media was the television. The television was found to be one of the major sources of information in other western countries as well [12, 13, 22]. Additionally, the present results suggest that people with a sufficient corona-specific HL tend to inform themselves by using several different sources, as they report higher proportions of use of almost all information sources compared to people with problematic or inadequate corona-specific HL. This finding indicates that people with higher corona-specific HL levels seem to be more sensitized and critical regarding the reliability of information and therefore might check for trustworthiness by comprising different sources. Despite the progressive digital transformation and the nearly omnipresent information on corona-specific issues, the use of different information sources by the general population corresponds to previous findings reporting that the internet, social media and other digital sources are complementary to rather than substituting traditional information sources such as health professionals or newspapers [31]. Even though the majority of the population seems to be well-informed on the coronavirus and COVID-19, large parts are still confused by the flood of information and the potential threat [23], which could be another reason for the incorporation of several information sources by at least some part of the population.

The highest trust was placed in information received from health authorities and health professionals. The same was found in Germany, where health professionals and health authorities were mentioned as the most trustworthy sources of information [22]. Also previous to the COVID-19 pandemic, health professionals were mentioned as the most trustworthy source of health information [32]. Nevertheless, in Switzerland especially at the beginning of the pandemic, less than a half of the respondents sought information on COVID-19 directly from health authorities (42%) and less than a quarter from health professionals (22%). On the one hand, this can probably be explained by the fact that in spring 2020 non-urgent doctor’s and other health professionals’ visits were not regularly available. On the other hand, most information provided by the official authorities was primarily transmitted through television, especially during the peak of the pandemic, and thus could have led to a more frequent naming of television instead of health authorities.

People with sufficient corona-specific HL more often reported higher trust in all of the information sources. What seems to be even more important, however, is the finding that the difference between people with high versus low corona-specific HL with great trust in health authorities and health professionals is much higher than the difference between these two groups regarding social media or other non-official sources. This result is in line with previous studies showing that people with lower HL are more likely to distrust health professionals [16, 28]. Lower trust in health professionals has also been found to be associated with less favorable health behaviors, more symptoms and lower quality of life [33], whereas a trustful relationship can improve treatment outcomes [18]. Therefore, lower corona-specific HL and the associated lower trust in health professionals could lead to higher risk behavior regarding the compliance to the preventive measures and worse health outcomes. Hence, increasing corona-specific HL could lead to greater trust in health care professionals and therewith could represent a promising approach to enhance preventive behavior and health outcomes.

With regard to *general and social sources* of information, the findings of the PCA show that the German-speaking population in Switzerland has more trust in *general sources* than in *social sources*. For both types of information source the level of trust corresponded to the usage of these sources. Furthermore, respondents reporting higher overall and actual trust in both information sources showed less difficulties when dealing with corona-related health information and thus higher corona-specific HL. Additionally, people reporting higher overall and actual trust in *general sources* were more likely to show higher corona-specific HL compared to those who tended to trust *social sources* more. Even though the effect of trust in *general sources* compared to *social sources* on corona-specific HL is smaller regarding actual trust, it can be assumed that people who have a higher corona-specific HL give more trust to *general sources.* Therefore it seems to be more likely that they get access to reliable information and follow the preventative instructions of authorities, health professionals, scientists and the media, which are often considered to be trustworthy and correct. Thus, it may be assumed, that people with higher trust in *general sources* are more likely to trust official information sources and follow their instructions. However, it remains unclear, whether higher trust in *general sources* leads to higher HL or whether higher HL results in more trust in *general sources*. Nonetheless, it may be concluded that with increasing trust in *general sources* of health information, the handling of this information could get easier and lead to higher (corona-specific) HL. Therefore, trust seems to be an essential part of corona-specific as well as general HL and should be considered as an important aspect of the concept and the enhancement of HL, as already pointed out before by others [18].

### Strengths and limitations

This paper presents the first results on corona-specific HL among the German-speaking population of Switzerland. The novelty of this study, however, is not only the relation of HL with corona-specific information, but especially the investigation of the differences in the use of and trust in information sources and their relation to HL.

However, this study also comprises some limitations that may affect the generalization of the results. First, the data was collected by online surveys only. Therefore, it may be possible that some people who do not or rarely use the internet were excluded from this study. Furthermore, for those people it might be more difficult to deal with corona-specific information, and consequently, corona-specific HL of the population might even be overestimated. For further data collections it would be advisable to consider additional methods as well (telephone, personal interviews). Second, when interpreting the results, the time of the survey needs to be carefully considered. For example, the initial data was collected after the first lockdown and during the time of the first openings. In order to make more precise statements regarding the development of the corona-specific HL and potential influences, further regular surveys at different stages of the pandemic would be needed. Third, the present results are based on a self-assessment by the respondents, which carries the risk of reporting bias and social desirability. However, there seems to be no reason why participants should have misreported difficulties regarding the handling of health information. In addition, the present measurement instrument has been shown to be valid and is based on widely used instruments to assess general HL. Ultimately, the results presented in this publication refer to the data collected in the German-speaking Cantons of Switzerland only. Thus, the findings could be different in the French- and Italian-speaking regions, as they were for example affected differently by the COVID-19 pandemic.

## Conclusion

In a situation of an «infodemic» where there is a rapid spread of a huge amount of information, both valid and invalid [7], HL acquires particular importance. In order to behave appropriately in times of a pandemic, the population needs easy-to-access and easy-to-use information and consistent and comprehensible communication. Policy makers, health professionals, the scientific community and the media need to support the population by providing accessible, trustworthy, and quality-assured information. In addition, individuals should be empowered to distinguish between trustworthy health information and mis- and disinformation [10]. The according skills are particularly important since the internet is one of the more frequently used information sources and bears a high risk of mis- and disinformation. Moreover, HL is not only an individual resource, but a relational construct involving multiple actors. Accordingly, the extent of HL in the population is always to be understood as the subject of interactive processes between individuals and society [20]. As trust in information sources and HL seem to be related, further research should investigate this relationship more in depth, especially in view of future health crises and pandemics. By improving the communication of health-related information and preventative measures, the adherence to them and the consequent protection of public health could be secured.

## Data Availability

Participants of this study did not agree for their data to be shared publicly, so supporting data is not available.

## Appendix

**Table 12:** Trust in information sources

	*% Inhabitants of German-speaking Switzerland*												
*Information sources* ^*a*^	Spring 2020				Fall 2020				Winter 2020				*p-value*
	++	+	–	–	++	+	–	–	++	+	–	–	
Health professionals	41%	46%	9%	4%	40%	47%	10%	3%	42%	48%	7%	3%	*0.311*
Health authorities	41%	41%	12%	6%	42%	40%	12%	6%	47%	39%	10%	4%	*0.049*
Info-Hotline	34%	47%	12%	7%	37%	45%	14%	4%	44%	41%	10%	5%	*< 0.001*
Radio	17%	61%	17%	5%	15%	59%	22%	5%	13%	58%	23%	6%	*0.018*
Internet	16%	55%	24%	5%	17%	55%	24%	4%	16%	58%	21%	5%	*0. 409*
Television	13%	55%	25%	7%	14%	59%	22%	5%	18%	61%	16%	5%	*< 0.001*
Newspaper - Offline	13%	54%	27%	7%	10%	58%	27%	6%	8%	53%	30%	9%	*0.001*
News App	8%	50%	31%	11%	8%	46%	38%	8%	6%	43%	40%	11%	*< 0.001*
Family, friends, acquaintances	9%	44%	39%	8%	10%	47%	37%	6%	9%	49%	36%	6%	*0.216*
Social media	4%	10%	56%	30%	4%	15%	49%	32%	4%	20%	42%	34%	*0.005*

*a. Not all sources of information asked are reported in the table*

*++ = very trustworthy; + rather trustworthy; − = rather untrustworthy; −- = very untrustworthy*
